# Clinical validation and assessment of a modular fluorescent imaging system and algorithm for rapid detection and quantification of dental plaque

**DOI:** 10.1186/s12903-017-0472-4

**Published:** 2017-12-28

**Authors:** Keith Angelino, Pratik Shah, David A. Edlund, Mrinal Mohit, Gregory Yauney

**Affiliations:** 1 Department of Media Arts and Sciences, Massachusetts Institute of Technology, 75 Amherst Street, E14, Cambridge, MA 02139 USA; 2 Hampden Dental Care, 7425 West Hampden Avenue, Lakewood, CO 80227 USA

**Keywords:** Dental plaque, Fluorescence, Oral health, Oral imaging, Porphyrin, Segmentation

## Abstract

**Background:**

Significant numbers of adults and children have untreated plaque due to poor oral hygiene and consequently suffer from associate dental and systemic diseases.

**Methods:**

A handheld device equipped with 405 nm light-emitting diodes was constructed to examine the prevalence of red fluorescence signatures associated with dental plaque. This device was used for in vivo imaging of all four incisors and all four canines of twenty-eight consenting human subjects. The same areas were further imaged under white light illumination with a commercial image-processing based plaque-imaging device, and evaluated by a hygienist and dentist. A custom computer vision algorithm using pixel information was developed to calculate plaque coverage ratios ranging from 0 (no plaque) to 1 (complete plaque coverage) for images captured by both devices.

**Results:**

The algorithm calculated red fluorescence-based plaque coverage ratios ranging from 0.011 to 0.211 for the subjects imaged. Clinical assessment and statistical analyses of associated plaque ratios of the 405 nm device images indicated high sensitivity and specificity in detecting dental plaque by the experimental device compared to the commercial reference device.

**Conclusions:**

The low-cost and open source 405 nm device and the associated computer vision algorithm successfully captured red fluorescence signatures associated with dental plaque and demonstrated comparable performance to a commercially available device. Therefore, a proof of concept validation was provided for the construction and application of a sensitive cost-effective plaque-detecting device. A miniaturized mobile adaptable version of the device was also provided, together with and a step-by-step guide for device assembly and webhost the associated software, to facilitate open-source access to a cost-effective at-home, in-clinic oral care technology.

**Trial registration:**

ClinicalTrials.gov NCT03379337, December 19 2017. Retrospectively registered.

**Electronic supplementary material:**

The online version of this article (doi: 10.1186/s12903-017-0472-4) contains supplementary material, which is available to authorized users.

## Background

The dental plaque biofilm is comprised of a collection of microorganisms [1–3], the acids and other substances produced when metabolizing the starches and sugars found in food [4] and shed epithelial lining. If not routinely mitigated, plaque will lead to subsequent complications such as dental caries and gingivitis [5]. Microbial acids, which erode the enamel and dentin of the tooth and penetrate into the tooth pulp, are the primary cause of dental caries. Untreated plaque can mineralize and form calculus deposits (tartar), which are too hard for brushing alone to remove [6–8]. Plaque and resulting calculus that inflame the gingiva are the primary cause of gingivitis and periodontitis, where the gingiva and bone that provides structural support for the tooth is damaged, resulting in pockets of lost tissue [5]. Furthermore, studies have proposed systemic linkages between oral diseases and a range of systemic diseases [9]. There is evidence that the bleeding sites serve as entryways for pathogens into the bloodstream [10], possibly leading to secondary infections and the manifestation or compounding of conditions such as stroke, cardiovascular and coronary artery disease [11, 12], atherosclerosis [9, 11, 12], endocarditis [10–12], and a variety of respiratory ailments including pneumonia [9, 11]. Thus, there is significant prophylactic value in periodic detection and removal of dental plaque.

Feedback to correct brushing and flossing techniques is usually unavailable to patients between dentist visits. Contributing to unmet oral care needs are high rates of failed appointments in certain populations [13, 14]. Consequently, vast sections of the population, even in the developed world, do not have accessible solutions to monitor oral health. Plaque detecting agents are available to consumers, but new technologies may offer a simpler way to reveal the biofilm. One approach to rapidly and non-invasively detect plaque exploits the fluorescent properties of porphyrins, a family of organic biomolecules found in all living cells and archetypally a component of numerous biosynthesis pathways [15]. In the oral environment, some porphyrins are metabolic products of heterogeneous bacteria within plaque [16, 17]. The porphyrin base structure, porphin, is an aromatic compound which consists of four pyrrole rings linked via methane bridges. Porphyrins commonly have modifications to the periphery of the base structure depending on their intended purpose [18, 19]. Porphin and porphyrins exhibit strong absorption of light anywhere from the longer ultraviolet A (UVA) wavelengths to the visible blue border; such absorption is known as the Soret band, with peaks dependent on the specific compound [20]. The absorption spectrum can be further altered by complexing the molecule with a central metal ion [21]. Excitation in the Soret band prompts a π → π* orbital promotion; during fluorescence, most porphyrins, including those within plaque, will emit photons of red wavelengths [22–24].

While some oral bacterial species have demonstrated red fluorescence on their own [25], Van der Veen et al. demonstrated a significant increase of red fluorescence when *P. micros* and *P gingivalis* were cultured only within close proximity, suggesting a co-operative metabolic pathway between species for porphyrin biosynthesis [24]. This may support observations which imply that oral diseases such as periodontal disease are a manifestation of bacterial communities rather than select species acting individually [26, 27]. Other sources of porphyrin have been debated, especially in the context of cancers [28–31]. Overall, presence of the porphyrin may be an indicator of bacterial activity [16, 17], and its potential usefulness as a diagnostic tool for various oral conditions [32] motivates ongoing investigation [26, 27].

Research groups typically construct custom illumination setups when studying oral red fluorescence [17, 33–38]. The conventional setup is comprised of an excitation light source directed at a dental subject, with a visible light camera imaging the illuminated area. A spectrometer have alternatively been used in place or in addition to the camera for measuring the fluorescence emission [17, 33, 36–38]. The source light is typically a light-emitting diode (LED) or laser with an approximate 400 nm wavelength; alternate setups have used broadband bulbs equipped with selective optical filtering [24, 25, 28]. The dental subject is commonly an in vitro plaque sample or a culture of oral bacteria. The imaging camera or spectrometer is usually equipped with an optical filter to remove the source light wavelength. While often qualitatively compared to white light images, plaque images can be further post-processed using simple image processing and segmentation techniques [39–42].

The ACTEON SOPROCARE (ACTEON North America, Mount Laurel, New Jersey, United States of America) is an intraoral probe which can operate under three modes (plaque “PERIO” mode, caries “CARIO” mode, and white light “DAYLIGHT” mode). In plaque mode, the SOPROCARE operates by illuminating plaque with both 450 nm and white light, and then digitally embellishing the color of plaque-affected areas. Newly formed plaque appears white to grey, and older, mature plaque is colored yellow to orange. The Q-Ray™ System (Inspektor Research Systems, Amsterdam, The Netherlands) is another multimodal oral imaging platform which selectively targets plaque, caries, and white spots. During operation, two images are taken in rapid succession: one white light image, and then one image illuminated under an approximate peak wavelength of 405 nm. Light is selectively filtered to emphasize red fluorescence for plaque, or a loss of fluorescence in unsound enamel. Further analysis and pixel segmentation options are available in the software. The SOPROCARE [43, 44] and the Q-Ray™, have both been used in clinical research studies [24, 25, 31, 32, 39, 45–49].

Using simple optical principles we demonstrate the construction and validation of a plaque-imaging device, “Plaquefinder”. In a clinical study of twenty-eight consenting human subjects, the device successfully exposed areas of red fluorescence without disclosing agents or onboard imaging processing. A custom computer vision segmentation algorithm to highlight regions with plaque accumulation and calculate plaque coverage was developed and provided a clinically accurate representation of dental plaque. We provide step-by-step assembly and instructions for use for Plaquefinder and determination of plaque score using segmentation software. Additionally, the webhosted segmentation software can highlight red fluorescence in images captured with other devices based on similar principles.

## Methods

### Device construction

**Fig. 1:**
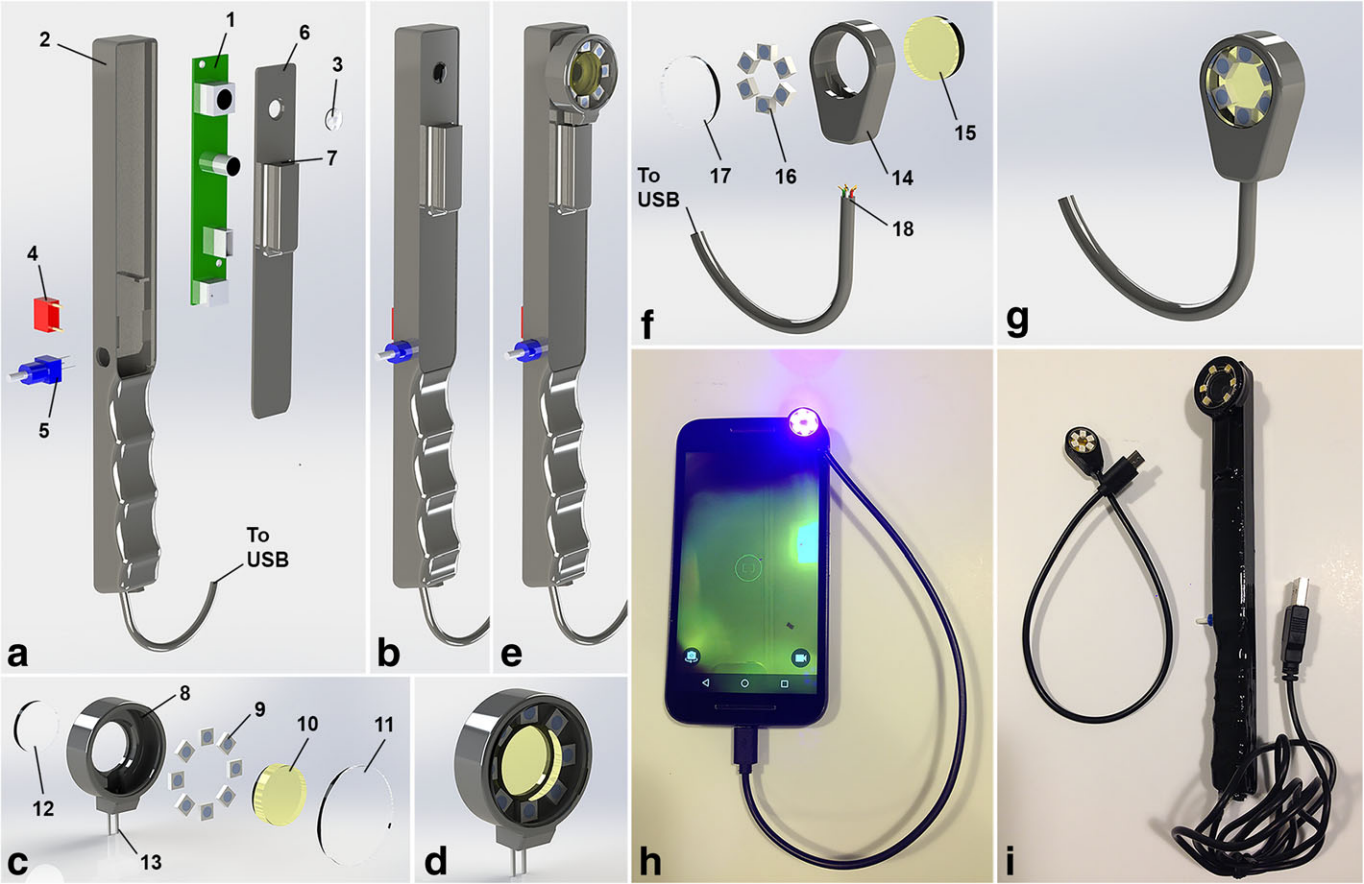
The Plaquefinder imaging device and its smartphone attachment variant. **a** device component view; **b** assembled view; **c** interchangeable head component view; **d** interchangeable head assembled view; **e** device with interchangeable head attached; **f** smartphone attachment component view; **g** smartphone attachment assembled view; **h** connected and powered smartphone attachment; **i** Plaquefinder smartphone attachment and original device (wire/electrical connections and joints not shown). Items A through G are CAD renderings

The Plaquefinder imaging device is comprised of an intraoral camera board with an attachment port for various interchangeable heads (Fig. 1a and b). The interchangeable heads (Fig. 1c and d) allow for modularity over different excitation light sources and optical filter combinations without the need for reconstructing the camera hand piece. The LED array within the interchangeable head could theoretically consist of any type or combination of emitter wavelengths within power constraints; we constructed arrays utilizing 405 nm emitters for porphyrin stimulation and a 530 nm cut-on filter to remove incident violet and light. White light interchangeable heads for visible light comparison were also constructed. A detailed description of the assembly as seen in Fig. 1 is available as an additional document [see Additional file 1]. We list the components and preferred vendors, stereolithographic (.STL) files for three-dimensionally printing the wand housing, and assembly instructions for building this version of the Plaquefinder on the World Wide Web at http://bit.ly/2rjwlYf. A computer or smartphone application, capable of running the USB (Universal Serial Bus)-connected camera, can display the live video on-screen.

Utilizing the same optics, the imaging device can be miniaturized from a handheld apparatus to a smartphone USB On-The-Go (OTG) attachment (Fig. 1f and g). Our version consists of a small custom plastic housing which contains the same 405 nm LEDs and filter. The circular LED array is placed concentrically around the frontward-facing camera aperture and secured, and the camera application started. The camera and powered LEDs are then positioned in front of the teeth while the user looks downward at the screen, using the camera viewfinder as the live feed.

### Clinical testing

The Massachusetts Institute of Technology’s Institutional Review Board, the Committee on the Use of Humans as Experimental Subjects, reviewed and approved the clinical protocol. Hampden Dental Care in Lakewood, Colorado was selected as the location for the trial. Data collection occurred from May to August 2016. Subjects aged 18–70 visiting the clinic site for dental prophylaxes, restoration assessment, or root canal treatment were invited to participate in the study. These individuals were notified of the study via a phone call or email 1 week prior to their appointment. During their actual visit, subjects were explained the details of the study and provided the opportunity to ask questions, refuse participation, or provide written consent. No compensation was offered for participation. Subjects exhibiting gingival bleeding or those undergoing oral surgery were also excluded from the study. Twenty-eight subjects consented within the study duration and passed exclusion criteria.

**Fig. 2:**
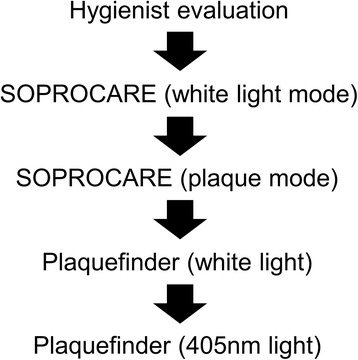
Image acquisition process during clinical study

The target teeth positions for imaging included all four incisors and all four canines. Only the vestibular and interproximal regions were of interest. Images were taken of these target teeth positions regardless of whether or not they exhibited plaque, consisted of full or partial restorations, contained major cracks/fractures, chips, or were missing. Other captured images included any abnormality of the tooth or gum exhibiting red fluorescence physically accessible by the device; images of locations outside of the target teeth were not included in the main analysis. Under the supervision of a practicing dentist, a dental hygienist rated the plaque of all target teeth under normal room lighting conditions (Fig. 2). Next, an Acteon SOPROCARE (herby referred to as the reference device) was used to take images of the target teeth in both white light and plaque modes. The SOPROCARE was chosen as it was the only plaque detection imaging device readily available within the United States and previouly evaluated in other published studies [43]. Afterwards, the Plaquefinder was used to capture images of all target teeth using the white light and then 405 nm interchangeable heads. A sweeping white light video of the vestibular side of the target teeth was also obtained for reference. A Hamamatsu C12880MA mini-spectrometer (Hamamatsu, Japan) was used to measure the wavelength of the red fluorescence in select instances to verify that the wavelength of the red fluorescence reflected parallel measurements in the established literature.

Apart from sex and age, no other subject demographic data was recorded. All images obtained by experimental plaque imaging and the reference device were de-identified at their time of collection to ensure patient confidentiality.

### Software and post-processing

A post-processing segmentation algorithm capable of automatically highlighting red fluorescence in 405 nm Plaquefinder images was developed. While various techniques which aim to segment dental plaque have been previously created [39–42], we use histogram thresholding for segmentation of images into plaque and non-plaque areas [50, 51]. Histogram thresholding is a general-purpose image processing algorithm that has previously been used for biofilm analysis [52] and digital mammography [53]. A comprehensive plaque coverage ratio for each subject was calculated using this algorithm from the aggregate of Plaquefinder images. The reference device plaque mode images colored identified plaque with a yellow/deep orange hue. These color signatures then were similarly segmented with threshold values readjusted to target yellow and deep orange hues using our segmentation algorithm to generate plaque coverage ratios. We characterized the reference device’s yellows in the L*a*b* color space and the Plaquefinder’s reds in the hue-saturation-value (HSV) colorspace, but either can be converted into the other color space. The thresholds for the reference device were selected as: L*: [0, 90]; a*: [−20, 13]; b*: [17, 78]. Plaquefinder thresholds were selected as: H: [0, 0.1] and [0.82, 1]; S: [0.15, 1]; V: [0.1, 1]. Plaque coverage ratios determined by images captured by Plaquefinder and the reference device were then compared. The web interface provides a demo where individual red fluorescence tooth images can be uploaded for segmentation and measurement.

## Results

Of the 28 subjects, 10 were female (mean age 47 years, range 31–69) and 18 were male (mean age 52 years, range 22–70). Of the target teeth imaged, 12 subjects had at least one major crack/fracture, 5 subjects had at least one major chip, 4 had at least one full restoration, and 2 had at least one partial restoration. No subjects had missing teeth. Red fluorescence was detected on teeth of 100% of the subjects with variable severity. The most common areas showing red fluorescence were along the gingiva and interproximal locations. Cracks, both naturally occurring and artificially formed (such as the margin between a restoration and residual tooth), frequently showed red fluorescence. Distributions on tooth facial surfaces were present but less common, usually only when the gingival margins and interproximal regions also had plaque.

**Fig. 3:**
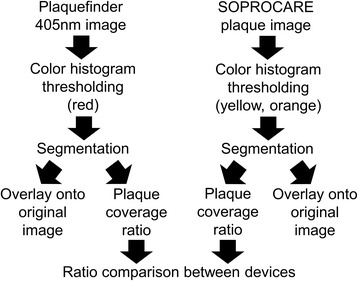
Image post-processing workflow for segmenting dental plaque

Each pixel captured in the image is represented by three values, one each for the amount of red, green, and blue in that pixel. These red, green, and blue values are normalized to ensure that they span the same range for each image. The values are too highly correlated to use directly in thresholding, so they are transformed into the HSV colorspace, which measures three different values in a cylindrical coordinate system for each pixel. We then designate as plaque all pixels that have HSV values within empirically determined thresholds that characterize the red color of plaque. Similarly, the numbers of uncovered tooth pixels are determined from their green fluorescence. We use this segmentation to calculate a plaque coverage ratio, derived from the ratio of the number of plaque pixels against the number of tooth pixels, as a measurement of how much plaque is visible on the teeth in an image (Fig. 3). Higher ratios indicate that the teeth in the photo are covered with more plaque. This plaque coverage ratio ranges from 0 (no plaque detected) to 1 (total tooth coverage of plaque).

Using the Plaquefinder device in red fluorescence mode, aggregate plaque coverage ratios of the subjects as determined by our software ranged from 0.011 to 0.211, with an average of 0.079. Review of the segmented plaque images against their ratios and hygienist ratings allowed for general numerical baselines to be created: 0–0.05 as excellent, 0.05–0.15 as fair, and >0.15 as poor. These baselines could serve as preliminary guides for prophylaxis recommendation; for instance, a ratio of 0.10 could correlate to a user instruction to increase brushing/flossing regimen. The same segmentation method was used on the reference device in plaque mode images to determine a plaque coverage ratio, and then the coverage ratios from the two devices were compared. Table 1 shows five comparative examples of plaque coverage ratios from select images between both devices; aggregate ratios for all subjects are available as an additional document [see Additional file 3].

**Table 1:** Computed plaque coverage ratios of the reference device and Plaquefinder for select images

Subject	Reference device (plaque mode)	Plaquefinder (red fluorescence)	Delta (Ref. device – Plaquefinder)
M2	0.4158	0.1891	0.2267
M3	0.3670	0.0213	0.3457
M18	0.2274	0.0418	0.1856
M24	0.4869	0.0099	0.4770
M100	0.3494	0.0561	0.2933

**Fig. 4:**
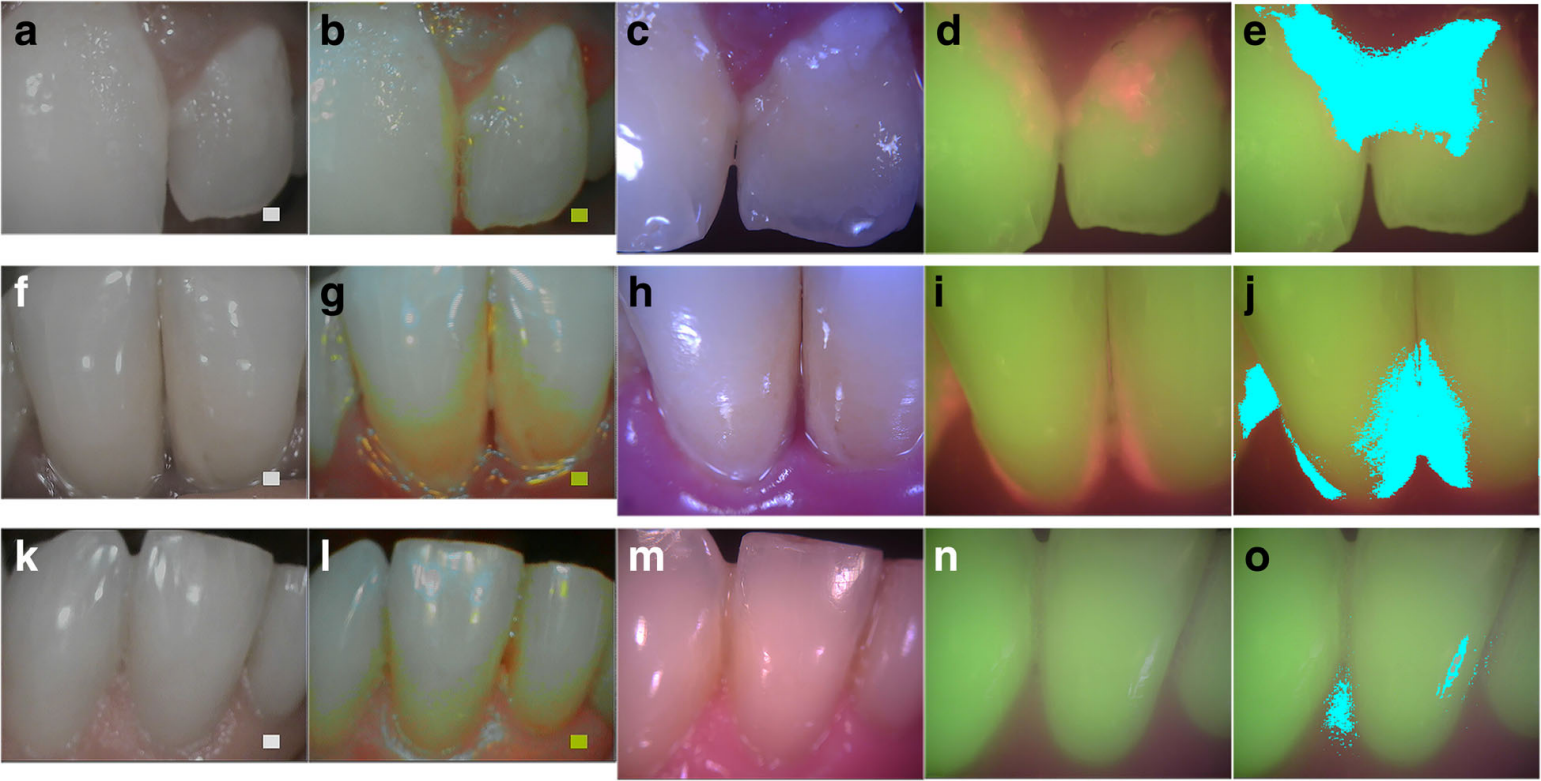
Comparison images captured by reference device and Plaquefinder. Columns from left to right: reference device white light; reference device plaque mode; Plaquefinder white light; Plaquefinder 405 nm light, segmented Plaquefinder 405 nm light images. In the Plaquefinder 405 nm images, teeth have a natural green fluorescence; plaque fluoresces red. Top row, **a**-**e**: plaque coverage of 0.2708 (M1). Middle row, F-J: plaque coverage of 0.1891 (M2). Bottom row, K-O: plaque coverage of 0.0213 (M3). Spectroscopic readings from M1 and M2’s red fluorescence are available in an additional document [see Additional file 2]

**Fig. 5:**
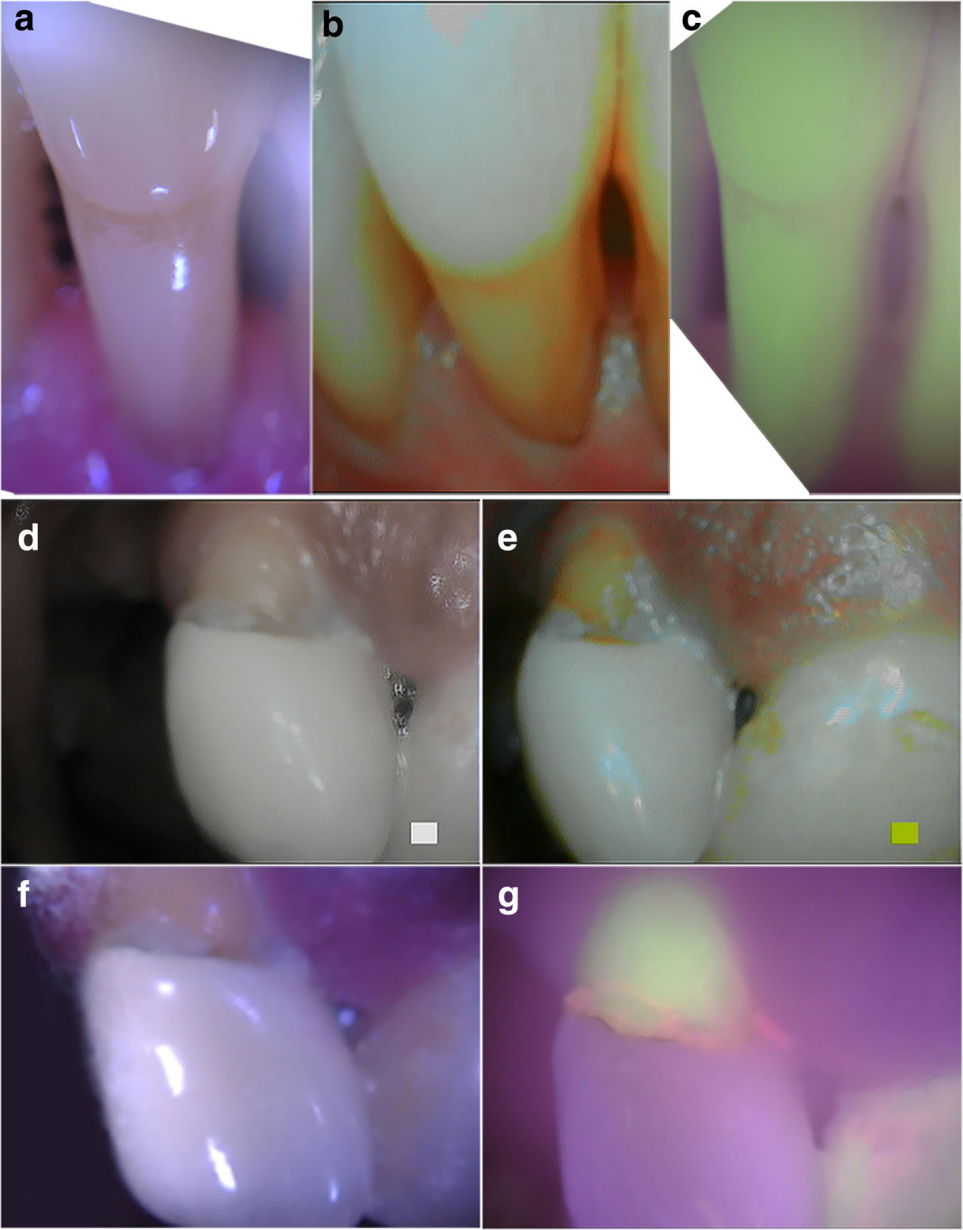
Special cases. Top row: **a** White light image of gingival recession; the dentin root of the tooth has been exposed. **b** The reference device exaggerated the natural yellow color of the dentin to look like plaque; however, (**c**) the Plaquefinder showed no visible red fluorescence. Hygienist confirmation of these teeth as clinically plaque-free demonstrates higher specificity in the Plaquefinder. Middle and bottom row: Dental restoration under different imaging modes: **d** Reference device white light; **e** Reference device plaque mode; **f** Plaquefinder white light; **g** Plaquefinder 405 nm light. The natural green fluorescence of the tooth is contrasted greatly from the restoration in (**g**). Bacterial activity is visible at the margin as indicated by red fluorescence, a common precursory location for secondary caries

Figure 4 compares images from the reference device and Plaquefinder from two subjects with confirmed plaque (M1 and M2) to a third participant exhibiting minimal plaque (M3). Newly-formed plaque imaged by the reference device in plaque mode appears clear and white, and was not significantly distinguished from white-light unaided visual identification without close scrutiny. This loss of discernibility is evident when comparing the reference device plaque image in Fig. 4b to the white light image of either device (Fig. 4a or 4c) in subject M1; in comparison, plaque is more visually representative in Fig. 4d. This coloration is visible in the reference device plaque images of M2 and M3 (Fig. 4g and l, respectively). The reference devices’ inaccurate identification of dentin as plaque is observable in instances such as during gingival recession. Fig. 5a-c demonstrates one such example; receded gingiva have exposed the dentin root (Fig. 5a **)**. The reference device has intensely enhanced the dentin to the same hues of mature plaque (Fig. 5b), but the Plaquefinder identifies no red fluorescence (Fig. 5c). The dental hygienist confirmed the teeth within the image to be clinically plaque-free, supporting the overall lack of red fluorescence. Plaquefinder identification of dental restorations was also accurate as fillings lacked the green autofluorescence of genuine teeth while under the 405 nm illumination. The reference device was not able to reliably distinguish restorations. Fig. 5d-g shows a restoration in differing imaging modes; under the 405 nm illumination, the residual tooth is easily contrasted from the nonfluorescent restoration (Fig. 5g).

## Discussion

This study provides an imaging device and analysis software effective in detecting plaque and determining plaque severity in a sensitive and accurate yet cost effective manner in comparison to conventional market device such as SOPROCARE. Consistent among reference device plaque images was yellow coloration along the gingival margin of variable width and hue, regardless of whether or not the teeth were determined to be plaque-free. Rechmann et al., attributes this yellow coloration to offsetting their plaque estimate [43]. This could potentially misdirect true plaque assessment, especially with inexperienced reference device users. Similarly, the reference device consistently estimated more plaque than red fluorescence alone; 21 out of the 28 subjects scored a higher plaque ratio with the reference device [see Additional file 3]. The overestimation and gingival margin exaggeration may be related to the reference device enhancing the natural yellow color of the root dentin, which is contrasted from the whiter enamel [54]. The enamel is progressively thinner towards the gingival margin and potentially more discernible to the reference device. Furthermore, enamel is generally thinner with age due to a longer period of erosion [55]. We postulate that as enamel loss and gingival recession increase with age, the reference device’s color enhancement techniques may incorrectly enhance the more prominent dentin in older patients and color them as mature plaque. Further inaccuracies by the reference device were hue exaggerations of interproximal spaces; gaps between teeth could appear deep orange or red, even if they were devoid of plaque (Figs. 4 and 5). Red fluorescence present at the margin between the tooth stump and restorations was also not labeled as plaque by the reference device (Fig. 5). Secondary caries remain a major cause of restoration replacement, confounded by difficulty in verifying the joint integrity [56]. The sensitivity of Plaquefinder in detecting plaque associated with dental restorations can serve as an additional aid for assessing the bacterial activity, coverage, and penetration.

The Plaquefinder and the reference device were limited by their size when attempting to access the more confined regions of the mouth. The use of segmentation for true plaque quantification is challenging without comprehensively imaging the mouth, including the posterior and lingual side of all teeth. A hypothetical method would be to capture red fluorescence data from a sweeping panorama or video of all buccal, lingual, and occlusal surfaces, with registration, knitting, and segmentation processes. This would require complete and consistent imaging of all dental surfaces and the removal of duplicate imaged regions, a task logistically difficult to achieve due to user inconsistency and the size of current intraoral cameras. We plan to conduct a future longitudinal study that correlates dental plaque to preexisting conditions such as gingivitis, which could be recognized with machine learning techniques. The study would also account for a greater number of tooth surfaces, as the prevalence of oral conditions is not homogenously distributed within the mouth [57]. Other associations to consider during a larger study include investigating the link between diet and plaque proliferation, which would build upon earlier work [58]. To further simplify the Plaquefinder, we repackaged the core optical components into a smartphone attachment. As described previously, this iteration has no internal camera, but is instead overlaid upon the smartphone’s frontward facing camera and allows rapid detection and segmentation of dental plaque without additional hardware.

## Conclusion

Plaque is a dental condition that, if left unattended, can lead to the proliferation of many oral diseases. While some market devices exist that image plaque, common impediments to widespread adoption are their price or the quality of their image processing. In a study of twenty-eight individuals, we demonstrate the affordable construction and validation of an imaging device that targets true red autofluorescence of porphyrin within plaque. A further simplification in design enables the Plaquefinder to be utilized as a smartphone attachment for at-home plaque detection and dispenses the onboard USB camera. By pursing hallmarks of active bacteria as an identifying marker, we eliminate factors such as stains and dentin coloration that confound other plaque segmentation methods. We also describe a free plaque segmentation algorithm to rapidly screen dental images with red fluorescence and provide a score of plaque coverage on teeth. The webhosted step-by-step guide for device assembly and associated software facilitate an in-clinic and in-home digital, low-cost and portable oral health screening system powered by minimal optics and image processing. While an experienced hygienist may identify majority of plaque without imaging devices, Plaquefinder can rapidly screen an inexperienced patient in the home environment or in outpatient settings at primary health physicians that usually offer no dental screenings.

## Additional files

Additional file 1: Device construction. Component description of Plaquefinder and smartphone attachment variant. (DOCX 13 kb)

Additional file 2: Spectroscopic readings of select subjects, Spectroscopic readings of select subjects in Fig. for red fluorescence wavelength approximation. Spectroscopic readings of plaque from Fig. 4a subject M1; (b) subject M2. Note multiple peaks in both subjects. M1 has at least three discernable peaks, with the most intense at approximately 640 nm. M2 has at least two discernable peaks, with the most intense also at approximately 640 nm. Cut-on filter at 530 nm. (DOCX 210 kb)

Additional file 3: Spectroscopic readings of select subjects, Spectroscopic readings of select subjects in Fig. for red fluorescence wavelength approximation. A positive Delta denotes the reference device as having detected more plaque, while a negative Delta denotes the Plaquefinder as having found more plaque. (DOCX 14 kb)

## Abbreviations

HSV: Hue-saturation-value
LED: Light-emitting diode
STL: Stereolithography
USB OTG: Universal Serial Bus On-The-Go
USB: Universal Serial Bus
UVA: Ultraviolet A
