# Supplementary material for: Clinical validation and assessment of a modular fluorescent imaging system and algorithm for rapid detection and quantification of dental plaque
Source: BMC Oral Health. 2017 Dec 28;17:162. doi: 10.1186/s12903-017-0472-4 (PMC5745686; doi:10.1186/s12903-017-0472-4)
Supplement: Supplementary file 1 — Device construction. Component description of Plaquefinder and smartphone attachment variant. (DOCX 13 kb) [file 12903_2017_472_MOESM1_ESM.docx]

DEVICE CONSTRUCTION: PLAQUEFINDER AND INTERCHANGEABLE HEAD

The wand portion in **Fig. 1A** and **1B** is comprised of a USB-connected camera (1) encased within a custom chassis (2), and fitted with a magnifying lens (3) over its aperture. An on/off switch (4) and a rotary dimmer switch (5) are also embedded in the chassis. A custom lid to the chassis (6) seats the magnifying lens and encases the internal circuitry. The lid also has an access point (7) by which the interchangeable heads (discussed below) are connected and powered. The end of the handgrip on the chassis has a hole in which the USB cable for the camera can pass. Interchangeable heads in **Fig. 1C** and **1D** have: a small, round chassis (8) seats a circular 405nm LED array (9) to evenly illuminate the target around the camera aperture. A 530nm cut-on optical filter (10) is placed in the center of the 405nm LED array to remove excitation light received by the camera, and then two protective glass windows (11, 12) encase and protect the LEDs and filter. Two terminals (13) friction fit into the access point mentioned previously in **Fig. 1A** and power the LED array. A white-light interchangeable head variant consists of a circular array of white LEDs and no filter assembled in the same housing. The USB cable to power the camera and LEDs exits through the bottom end of the chassis at the handgrip. Once assembled, all custom casings were sealed with an FDA-compliant hard epoxy resin. The entire assembly procedure, prior to epoxy sealing, typically takes less than three hours and can be performed by a minimally skilled operator. To connect the interchangeable head to the main wand, the head terminals are inserted into the access point as shown in **Fig. 1E**.

DEVICE CONSTRUCTION: PLAQUEFINDER SMARTPHONE ATTACHMENT

The smartphone attachment variant **(Fig. 1F and 1G)** consists of a small custom plastic housing (14) that fits a 530nm cut-on optical filter (15), a circular 405nm LED array (16), and a protective glass window (17). At the base of a chassis is a hole for a stripped USB cable to enter (18); the cable must have USB OTG capability in order to supply power the LEDs. Once components are secured, the device is sealed with an FDA-compliant hard epoxy resin. To use the smartphone attachment, the device is inserted into a smartphone that supports USB OTG (**Fig. 1H**). The frontward-facing camera is intended for use with this attachment. The circular LED array is placed concentrically around the frontward-facing camera aperture and secured, and the camera application started. The camera and powered LEDs are then positioned in front of the teeth while the user looks downward at the screen, using the camera viewfinder as the live feed. Not all smartphones support USB OTG; additionally, the camera must be capable of focusing on the teeth within the order of centimeters in order to capture the fluorescence signal.
