# Supplementary figures and images for: Clinical validation and assessment of a modular fluorescent imaging system and algorithm for rapid detection and quantification of dental plaque
Source: BMC Oral Health. 2017 Dec 28;17:162. doi: 10.1186/s12903-017-0472-4 (PMC5745686; doi:10.1186/s12903-017-0472-4)

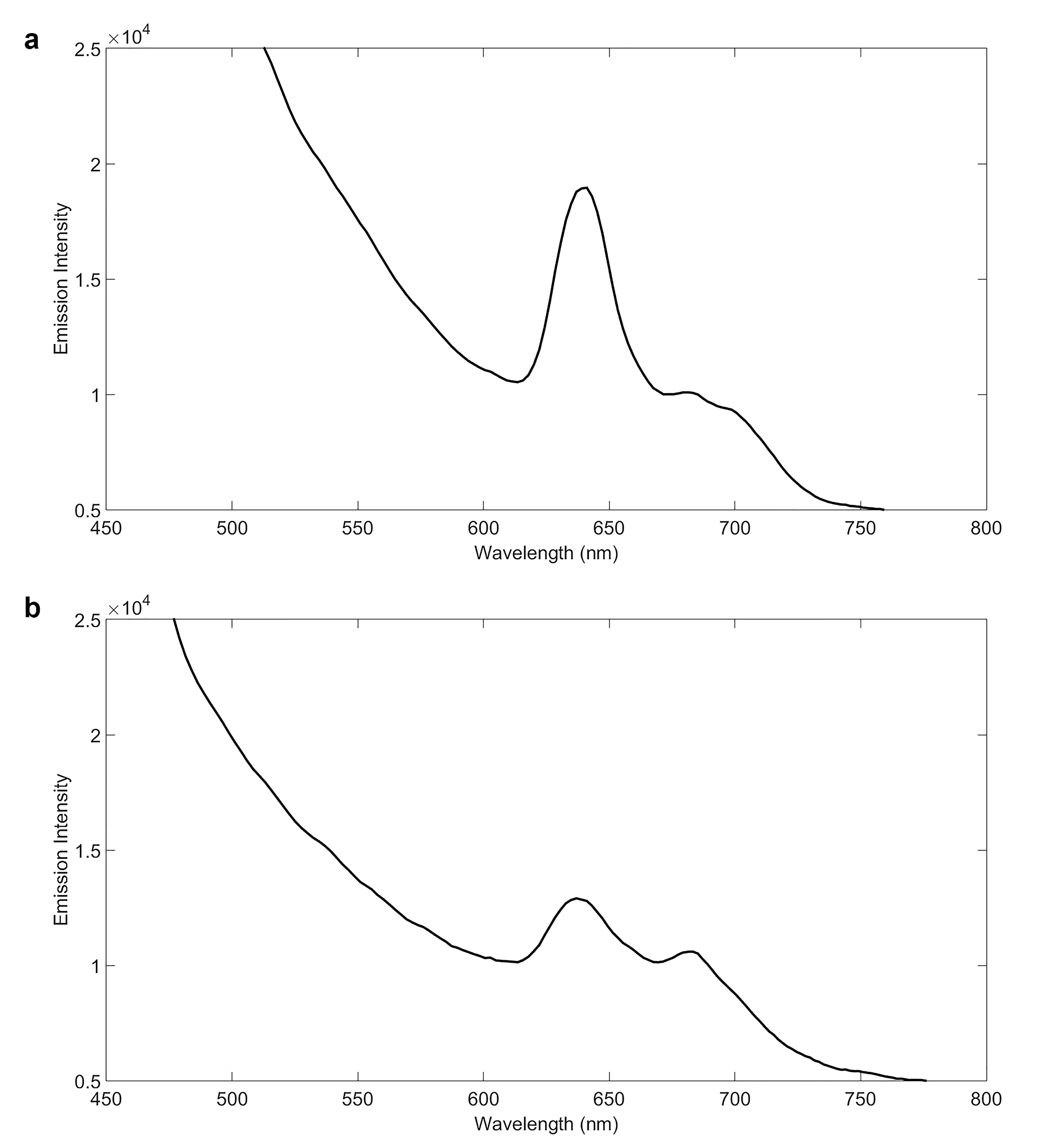

Supplement: Supplementary file 2 — Spectroscopic readings of select subjects, Spectroscopic readings of select subjects in Fig. for red fluorescence wavelength approximation. Spectroscopic readings of plaque from Fig. 4a subject M1; (b) subject M2. Note multiple peaks in both subjects. M1 has at least three discernable peaks, with the most intense at approximately 640 nm. M2 has at least two discernable peaks, with the most intense also at approximately 640 nm. Cut-on filter at 530 nm. (DOCX 210 kb) [file 12903_2017_472_MOESM2_ESM.docx]
