# Supplementary material for: Clinical validation and assessment of a modular fluorescent imaging system and algorithm for rapid detection and quantification of dental plaque
Source: BMC Oral Health. 2017 Dec 28;17:162. doi: 10.1186/s12903-017-0472-4 (PMC5745686; doi:10.1186/s12903-017-0472-4)
Supplement: Supplementary file 3 — Spectroscopic readings of select subjects, Spectroscopic readings of select subjects in Fig. for red fluorescence wavelength approximation. A positive Delta denotes the reference device as having detected more plaque, while a negative Delta denotes the Plaquefinder as having found more plaque. (DOCX 14 kb) [file 12903_2017_472_MOESM3_ESM.docx]

| Subject | Reference device (plaque mode) | Plaquefinder (red fluorescence) | Delta (Ref. device – Plaquefinder) |
| --- | --- | --- | --- |
| M1 | 0.3097 | 0.069 | 0.2407 |
| M2 | 0.1985 | 0.1868 | 0.0117 |
| M3 | 0.0358 | 0.1351 | -0.0993 |
| M4 | 0.1256 | 0.0254 | 0.1002 |
| M5 | 0.139 | 0.1425 | -0.0035 |
| M7 | 0.0785 | 0.092 | -0.0135 |
| M9 | 0.0653 | 0.015 | 0.0503 |
| M10 | 0.001 | 0.1358 | -0.1348 |
| M12 | 0.1112 | 0.0242 | 0.087 |
| M14 | 0.18 | 0.0562 | 0.1238 |
| M15 | 0.0451 | 0.0316 | 0.0135 |
| M16 | 0.1425 | 0.0362 | 0.1063 |
| M18 | 0.0464 | 0.038 | 0.0084 |
| M19 | 0.1723 | 0.0111 | 0.1612 |
| M20 | 0.1918 | 0.211 | -0.0192 |
| M22 | 0.1781 | 0.0535 | 0.1246 |
| M24 | 0.1288 | 0.0493 | 0.0795 |
| M25 | 0.1901 | 0.0201 | 0.17 |
| M26 | 0.0998 | 0.1233 | -0.0235 |
| M30 | 0.0277 | 0.0767 | -0.049 |
| M49 | 0.0772 | 0.031 | 0.0462 |
| M51 | 0.0502 | 0.0036 | 0.0466 |
| M59 | 0.2423 | 0.0701 | 0.1722 |
| M78 | 0.12 | 0.0719 | 0.0481 |
| M80 | 0.0693 | 0.0362 | 0.0331 |
| M88 | 0.1641 | 0.0424 | 0.1217 |
| M100 | 0.3052 | 0.2051 | 0.1001 |
| M105 | 0.0817 | 0.207 | -0.1253 |
